# Editorial: Bacteria-phage coevolution in antimicrobial resistance

**DOI:** 10.3389/fmicb.2023.1221867

**Published:** 2023-06-15

**Authors:** Pingfeng Yu, Hanpeng Liao, Na Wang, Ramesh Goel

**Affiliations:** ^1^College of Environmental and Resource Sciences, Zhejiang University, Hangzhou, China; ^2^Fujian Provincial Key Laboratory of Soil Environmental Health and Regulation, College of Resources and Environment, Fujian Agriculture and Forestry University, Fuzhou, China; ^3^Nanjing Institute of Environmental Sciences, Ministry of Environmental Protection, Nanjing, China; ^4^Department of Civil and Environmental Engineering, University of Utah, Salt Lake City, UT, United States

**Keywords:** antimicrobial resistance, antimicrobial resistance gene, phage, phage therapy, bacteria-phage coevolution

Antimicrobial resistance (AMR) poses a severe threat to global public health as it undermines the effectiveness of antimicrobial therapy for infectious diseases. AMR is driven by complex interactions of multiple factors, including overuse and misuse of antimicrobial drugs, poor infection control practices, and environmental pollution. With the global spread of resistant bacteria and increasing understanding of phages, the use of phages in controlling environmental antibiotic resistance contamination and treating antibiotic-resistant bacterial infections is receiving increasing attention. Phages, which are viruses that can infect bacteria, have long been considered potential alternatives to antimicrobials as they can selectively target and kill host bacteria without affecting good bacteria. Therefore, they can act as a biological control agent for bacteria-induced animal, plant, and human diseases.

The main aim of this Research Topic is to reveal the application of bacteria-phage coevolution in antibiotic resistance to better refine phage therapies. Within this theme, five articles have been published that complement our knowledge of phage therapy and the transfer of antimicrobial resistance genes (ARGs).

Recent studies have demonstrated the potential of phage therapy as an alternative to antibiotics for treating bacterial infections, in addition to the use of endolysins (phage-encoded peptidoglycan hydrolases responsible for bacterial lysis). *Streptococci* cause many diseases from common streptococcal pharyngitis to life-threatening severe conditions such as pneumonia and meningitis. Wong et al. highlighted the types of catalytic and cell wall-binding structural domains found in streptococcal endolysins and provide a comprehensive overview of the lytic capabilities of natural and engineered streptococcal endolysins studied to date and their potential applications in different industries. They conclude that the high recognition ability and binding specificity of endolysins can vary from entire bacterial genera to specific bacterial strains, and endolysins have minimal side effects on the normal microbiota, making them a good candidate for alternative antibiotic therapy.

Feng et al. used *Klebsiella pneumoniae* strain SXFY507 as an indicator bacterium and isolated a lytic phage vB _ KpnS _ SXFY507 from hospital wastewater. The physiological characteristics and genome of the phage vB _ KpnS _ SXFY507 were studied and analyzed, and the sterilization effect of the phage vB _ KpnS _ SXFY507 on the strain SXFY507 was investigated by the *in vitro* culture method. The results showed that phage vB _ KpnS _ SXFY507 had a short latency of 20 min and a giant mutant of 246 phages/cell. The phage vB _ KpnS _ SXFY507 has a wide host range, broad pH tolerance, and high thermal stability. The phage vB _ KpnS _ SXFY507 has a genome length of 53,122 bp, a G + C content of 49.1%, 81 open reading frames (ORFs), no genes involved in virulence or antibiotic resistance, and significant antibacterial activity *in vitro*. The survival rate of greater wax borer larvae inoculated with *Klebsiella pneumoniae* (*K. pneumoniae*) sxfy507 was 20%. In contrast, the survival rate of *K. pneumoniae-*infected larvae increased from 20% to 60% within 72 h after treatment with the phage vB _ KpnS _ SXFY507. These findings suggest that phage vB _ KpnS _ SXFY507 has the potential as an antimicrobial agent to control *K. pneumoniae*.

*Pseudomonas aeruginosa* (*P. aeruginosa*) is a significant cause of acute and chronic infections (e.g., urinary tract infections, burn skin infections, and lung infections). It is a common health problem in clinical practice. *P. aeruginosa* phages can effectively inactivate highly stable multidrug-resistant *P. aeruginosa* and are easy to administer. Therefore, to develop efficient *P. aeruginosa* phages, two new *P. aeruginosa* phages, named PPAY and PPAT, were isolated and characterized from wastewater treatment plants by Yuanyuan et al.. Their biological and genomic properties were investigated, and it was found that PPAT and PPAY had many similarities, but they differed significantly in terms of spotting, morphology, and growth characteristics. These two new isolates could be used for phage therapy and are promising tools for controlling *P. aeruginosa*.

The combination of fecal microbiota transplantation (FMT) and a phage mixture was used by Wang et al. to observe the therapeutic effect on *Salmonella typhimurium*-induced colitis in mice. The results showed that the combination treatment eliminated *S. typhimurium* after 72 h of treatment and effectively reversed the symptoms of colitis, which was superior to single phage or single FMT treatment. Gut microbiota analysis also showed that combining phage mixture and FMT effectively restored gut microbial diversity. In addition, PcFMT treatment significantly increased the levels of short-chain fatty acids (SCFA). In this combination therapy, the phage mixture and FMT acted as a “pathogen eliminator” and a “metabolic enhancer,” respectively.

Despite these promising results, bacteria are equally prone to develop resistance to phage during phage therapy, which significantly hinders the development of phage therapy for drug-resistant bacteria. Exploring the mechanisms by which bacteria rapidly produce phage resistance and overcoming bacterial resistance to phages has become a primary issue in phage therapy for bacterial infections. Unlike antibiotics, phages and microorganisms can also evolve in various ways to restore bactericidal activity to bacteria resistant to phages. This phenomenon is known as the coevolution of phages with their host bacteria. Understanding the coevolution between bacteria and phages can provide new insights into designing new antimicrobial strategies and controlling bacterial infections. Further research is needed to fully understand the potential of bacteria-phage coevolution in the fight against AMR. As the relationship between phage and bacteria is becoming better studied and understood, this relationship will become an essential solution to the problem of antibiotic resistance.

Soil is one of the most biologically active spheres on the earth's surface and one of the most relevant environmental elements for humans. As human activity increases (reclaimed water irrigation, organic fertilizer farming, etc.), soil contamination with antibiotic resistance increases. Soil has become an essential reservoir of antibiotic-resistant bacteria, resistance genes, and an important hotspot for the horizontal transfer of ARGs. Zhang et al. reviewed the progress of research on phages as vectors of ARGs in soil and analyzed the contribution of phages to the horizontal transfer of ARGs. They concluded that ARGs could be transferred horizontally at a high transduction frequency, including specialized, broad, and lateral transduction. Transduction can occur without contact between donor and recipient bacteria and is not limited by time or space. Human activities, particularly the application of organic fertilizers, can lead to the enrichment and spread of ARGs in soil-borne phages. Therefore, the role of phages in ARG transfer should be considered.

Overall, while there are still some challenges that need to be addressed regarding phage biocontrol, the results are encouraging and bring hope for combating global AMR.

## Author contributions

PY wrote the initial draft of the manuscript while HL, NW, and RG provided substantial critical feedback and editing. All authors contributed to the article and approved the submitted version.

